# Insomnia Complaints and Perceived Immune Fitness in Young Adults with and without Self-Reported Impaired Wound Healing

**DOI:** 10.3390/medicina58081049

**Published:** 2022-08-04

**Authors:** Jessica Balikji, Maarten M. Hoogbergen, Johan Garssen, Thomas Roth, Joris C. Verster

**Affiliations:** 1 Division of Pharmacology, Utrecht Institute for Pharmaceutical Sciences, Utrecht University, 3584 CG Utrecht, The Netherlands; 2 Division of Plastic Surgery, Catharina Ziekenhuis, Michelangelolaan 2, 5623 EJ Eindhoven, The Netherlands; 3 Global Centre of Excellence Immunology, Nutricia Danone Research, 3584 CT Utrecht, The Netherlands; 4 Sleep Disorders & Research Centre, Henry Ford Hospital, Detroit, MI 48202, USA; 5 Centre for Human Psychopharmacology, Swinburne University, Melbourne, VIC 3122, Australia

**Keywords:** insomnia, sleep complaints, fatigue, perceived immune fitness, wound healing, wound infection, slow healing wounds

## Abstract

*Background and Objectives*: Adequate sleep and an effective immune system are both essential to maintain a good health status. The current study aimed to determine the nature of insomnia complaints and perceived immune fitness among Dutch young adults with and without self-reported impaired wound healing. *Materials and Methods*: A total of (*n* = 2033) Dutch students (83.8% women) completed an online survey. Perceived immune fitness was assessed with a single-item scale and insomnia complaints with the SLEEP-50 insomnia subscale. The sample comprised a control group without self-reported impaired wound healing (*n* = 1622), a wound infection (WI) group (*n* = 69), a slow healing wounds (SHW) group (*n* = 250), and a COMBI group that experienced both WI and SHW (*n* = 92). *Results*: Comparisons with the control group revealed that individuals of the SHW and COMBI groups reported significantly poorer perceived immune functioning, increased insomnia complaints and daytime fatigue, and poorer sleep quality. *Conclusions*: Individuals with self-reported impaired wound healing have a poorer perceived immune functioning, increased insomnia complaints, daytime fatigue, and poorer sleep quality.

## 1. Introduction

A bidirectional relationship has been demonstrated between sleep and immune functioning [1,2,3,4,5,6]. Adequate sleep and an effective immune system are both essential to maintain a good health status. Studies have shown that sleep loss is related to reduced immune functioning and thus increases the susceptibility to disease [7]. Conversely, sleep quality and total sleep time are compromised during infection [8,9]. Sleep is also important for skin homeostasis, and insufficient sleep interferes with the barrier function of the skin [10,11]. Sleep loss has significant effects on protein synthesis, cell division, and growth hormone release, as these processes occur during sleep [12]. Consequently, sleep loss can negatively impact wound healing.

Wound healing progresses through three overlapping phases, including acute inflammation, proliferation and granulation tissue formation, and tissue remodeling [13,14]. The immune system is involved each of these phases. The physiological integrity of the skin is extremely important for maintaining psychological and physical health and so is normal sleep [15,16]. Enforced by the demands of our 24-h society [17], the average duration of a normal night of sleep (seven to eight hours) has been reduced by 1.5–2 h per night over the past decades [18]. Sleep (loss) has an important impact on various aspects of the wound healing process, and impaired wound healing has been associated with poorer perceived immune fitness, reduced mood, and poorer quality of life [19,20].

Research has revealed that sleep loss is related to the dysregulation of immune functioning. For example, in rats, sleep deprivation leads to hormonal [21,22,23], metabolic [23,24,25], neurochemical alterations, and impacts the immune system [26]. Consequently, sleep loss has adverse effects on the skin and mucosal barrier functions in rodents, as they acquire noninfectious ulcerative and hyperkeratotic lesions on their paws and tails [27,28,29,30,31].

In humans, sleep loss may also negatively affect the skin barrier function [32,33]. For example, in healthy men, Dimitrov et al. [24] demonstrated a significant decrease in tumor necrosis factor (TNF-α) levels while sleeping compared to being awake at night, and Ruiz et al. [25] observed an increase in levels of leukocytes, neutrophils, and cluster of differentiation 4 (CD4^+^) during forty-eight hours of total sleep deprivation. Alterations in host defense functions might be caused by sleep loss-induced changes in proinflammatory cytokines, as this can lead to a decrease in skin function recovery. However, other studies did not report changes in the interleukins (IL-1β and IL-6), and TNF-α in relation to sleep [26,27,28]. In addition, increased levels of C-reactive protein (CRP) were found in patients with sleep loss, which are also present during inflammation [31,34]. These higher levels returned to baseline levels after effective treatment [35].

Taken together, these studies demonstrated that the immune system plays an essential role in both sleep and wound healing. It is therefore important to further assess the relationship between sleep and wound healing. Insomnia, the most common sleep disorder, is defined as a sleep–wake disorder where the predominant complaint is dissatisfaction with the sleep quantity of quality. Insomnia is associated with one (or more) or the following symptoms: (1) difficulty initiating sleep, (2) difficulty maintaining sleep, characterized by frequent awakenings or problems returning to sleep after awakenings, and (3) early morning awakenings with an inability to return to sleep [36]. To the best of our knowledge, data relating insomnia to wound healing are lacking. Given the association of impaired wound healing with reduced perceived immune functioning and poorer mood [19,20], it is hypothesized that insomnia complaints will be more prevalent among individuals with impaired wound healing. To further investigate this, the aim of this study was to evaluate the relationship between perceived immune functioning, insomnia, and daytime alertness.

## 2. Methods

A sample of 18–30-year-old Dutch university students participated in an online survey on food and health. SurveyMonkey was used to design the survey. Participants were recruited via Facebook. The University of Groningen Psychology Ethics Committee approved the study (Approval code: 16072-O, approval date: 25 October 2016). Electronic informed consent was obtained from all participants.

In addition to demographics, participants could indicate whether they had experienced slow-healing wounds or wound infection during the past year. Based on their answer, participants were allocated to either (1) a control group (participants without slow-healing wounds or wound infection), (2) a wound infection (WI) group, (3) a slow-healing wounds (SHW) group, or (4) a COMBI group (participants reporting both WI and SHW).

Perceived immune fitness was rated on a single-item scale, ranging from 0 (very poor) to 10 (excellent) [37,38]. Insomnia was assessed with the 9-item SLEEP-50 insomnia subscale [39]. Each item of the SLEEP-50 can be scored on a 4-point scale, with the answering possibilities “not at all” (score 1), “somewhat” (score 2), “rather much” (score 3), and “very much” (score 4). The total insomnia score is computed by adding together scores on the individual items. Spoormaker et al. [39] considered a total insomnia score ≥19 as a positive screen for insomnia. In addition to the SLEEP-50 insomnia subscale, participants reported their average time to bed, time to start sleeping, and wake up time. Using this data, the total sleep time (TST) and sleep onset latency (SOL) were computed. Participants also reported their average number of nightly awakenings. Finally, sleep quality was scored on a scale ranging from 0 (very bad) to 10 (very good) [40]. Two subscales of the profile of mood states—short form (POMS-SF) questionnaire [41,42] were completed to assess fatigue and vigor (energy and alertness). The individual items were scored on a 5-point Likert scale (0 = not at all to 4 = extremely), and a sum score for the two scales was computed. Higher sum scores imply more fatigue or more vigor, respectively.

SPSS (IBM Corp. Released 2013. IBM SPSS Statistics for Windows, Version 28.0, Armonk, NY, USA: IBM Corp.) was used to conduct the statistical analyses. Nonparametric tests were conducted, as the data were not normally distributed. The independent samples Kruskal–Wallis test was used to conduct comparisons between the four groups (control, SHW, WI, and COMBI). To account for multiple comparisons, a Bonferroni’s correction was applied. Differences between groups were considered significant if *p* < 0.0083 (*p* < 0.05/6 comparisons) A chi-square test was used to compare percentual data. Again, a Bonferroni’s correction was applied. Finally, Spearman’s correlations were computed between perceived immune fitness and sleep outcomes. Applying a Bonferroni’s correction, the *p*-value for significance was set at *p* < 0.0071 (*p* < 0.05/7 correlations).

## 3. Results

A total of (*n* = 2033) participants (83.8% women) completed the survey. Their demographics are summarized in Table 1. Perceived immune fitness of the SHW group (*p* < 0.001) and COMBI group (*p* < 0.001) was significantly lower than the control group. No significant difference was found between perceived immune fitness of the WI and control group (*p* = 0.124) or other pairwise comparisons for this variable. No significant differences were found between the groups for the other variables.

The sleep characteristics of the four groups are summarized in Table 2. The sleep quality was significantly poorer in the SHW group (*p* < 0.001) and COMBI group (*p* = 0.002) compared to the control group. The sleep quality of both the COMBI group (*p* = 0.007) and the SHW group (*p* = 0.007) were also significantly poorer than the WI group. No significant differences between the impaired wound healing groups and the control group were observed for the total sleep time, nightly awakenings, and sleep onset latency. Pairwise comparisons between the wound healing groups also revealed no significant differences.

Table 3 summarizes the data of the SLEEP-50 insomnia scale. Compared to the placebo group, the total insomnia scores were significantly higher for both the SHW group (*p* < 0.001) and the COMBI group (*p* < 0.001). The total insomnia score of the WI group did not differ significantly from the control group. Pairwise comparisons between the impaired wound healing groups did not reveal significant differences.

With regard to individual items, pairwise comparisons revealed that the scores of the WI group did not significantly differ from the control group for any item. In contrast, the SHW group scores were significantly higher than the control group scores on all items, except for the item assessing sleep maintenance (“I wake up early and cannot get back to sleep”). The highest scores were found for the COMBI group. For this group, the scores on all items were significantly higher than those of the control group, except for the item addressing whether the total sleep time experienced is sufficient (“I sleep too little”). Pairwise comparisons further showed that, compared to the WI group, the COMBI group scored significantly higher on the item “I worry and find it hard to relax” (*p* = 0.009). Differences in the other items between the impaired wound healing groups were not significant. Together, these findings suggest that both WI and SHW, but especially their combination, are associated with an increased focused attention on distressing issues, resulting in worrying and rumination that may negatively affect sleep.

Table 4 summarizes the assessments of daytime fatigue and vigor. Compared to the control group, significantly increased fatigue scores were reported by the SHW group (*p* < 0.001) and the COMBI group (*p* < 0.001). Compared to the control group, significantly reduced vigor scores were reported by the SHW group (*p* < 0.001) and the COMBI group (*p* < 0.001). Together, these findings suggest that increased insomnia complaints among the SHW and COMBI groups are associated with increased daytime fatigue and reduced physical activity and vigor.

Table 5 lists the Spearman’s correlations between perceived immune fitness and sleep outcomes. Except for the total sleep time, all correlations were statistically significant.

## 4. Discussion

The association between slow-healing wounds or wound infections, sleep, and perceived immune functioning is extremely important. Both good sleep quality and adequate immune function are essential to maintaining health. This study demonstrated that young adults with self-reported impaired wound healing experience significantly more insomnia complaints. Participants with slow-healing wounds or wound infections scored significantly higher on the SLEEP-50 insomnia subscale and reported a significantly poorer sleep quality than the control group of participants without impaired wound healing. Individuals of the impaired wound healing groups screened significantly more often as positive for insomnia (31.9–45.2%) than the control group (25.8%). In our opinion, the observed difference of 5–20% in the percentage of individuals that screened positive for insomnia can be viewed as a clinically relevant difference. In addition, significantly higher scores of daytime fatigue were reported. In conjunction, perceived immune functioning was rated significantly poorer by the impaired wound healing groups, and the ratings correlated significantly with most sleep outcomes. Future intervention studies should confirm to what extent individuals with impaired wound healing will benefit from improving sleep quality and immune fitness. Taken together, the current study shows that insomnia-related sleep disturbances are associated with impaired wound healing.

To the best of our knowledge, no other studies have previously reported on the relationship between impaired wound healing and insomnia. However, other sleep disorders have been associated with impaired wound healing, and these findings are in line with the current study. For example, sleep complaints have been reported for patients with diabetic foot ulcers [43,44]. Diabetes mellitus is a disease resulting from impairment in insulin secretion, which is characterized by an increased level of blood glucose. About 15–25% of diabetic patients will develop chronic foot or lower extremity ulcers [45]. Patients with diabetic foot ulcer are also affected by lower sleep quality due to physical distress and impaired glucose metabolism [46,47,48] and manifested complications [49,50], such as nocturia, polyuria, diabetic neuropathy and neuropathy pain, and depression [51,52]. The delayed foot ulcer healing is a consequence of a reduced blood supply with subsequent hypoxia [53]. As a consequence, obstructive sleep apnea (OSA) is commonly reported among patients with diabetes, with or without foot ulcers [54,55,56,57]. A previous study [58] reported that pain due to the presence of leg ulcers negatively affected sleep. Since leg ulcers are often more painful in the afternoon or evening, this could therefore have an increased impact on sleep [59,60]. Other studies highlighted the high prevalence of sleep loss in chronic wound patients (58.8%) [61] and leg ulcer patients (69.0%) [62].

The limitations of the current study should be taken into account when interpreting the presented results. Firstly, the study was conducted among students aged 18–30 years old. It is not known to what extent our findings can be extrapolated to older age groups. Lifestyle may play a critical role in experiencing sleep complaints—in particular, among young adults [59]. Additionally, the sample was comprised of only students, and this may have influenced the study outcomes. Research has shown that students more often have irregular sleep patterns than their peers who have a regular nine-to-five job [20]. Furthermore, factors such as socioeconomic status, transition from living in a family setting to student life, alcohol consumption, and academic stressors may differ between students and non-students. Therefore, future research should verify our findings in non-student samples and older age groups. Secondly, females were overrepresented in the study. Although this reflects the sex distribution at the universities in the Netherlands, this may also have had an impact on the study outcome. That is, the literature shows that sleep complaints are more frequently reported by females compared to males [63,64]. Sex differences have also been reported for the prevalence of impaired wound healing [65]. Unfortunately, in the current study, the sample sizes of the WI, SHW, and COMBI groups were too small to allow well-powered analyses to further investigate potential sex differences. Future research should therefore aim at including larger samples with better balanced sex distributions. Thirdly, it should be realized that no formal criteria for insomnia or impaired wound healing were established in the current study. Allocation to the impaired wound healing groups or control group was entirely based on self-reports, without a formal diagnosis to support this. The SLEEP-50 was not developed to diagnose patients, but its use is intended as a first screening instrument [39]. In the current study, all assessments were based on self-reports. Additionally, perceived immune fitness, measured by single-item rating, is a subjective assessment. Although objective assessments of immune functioning (e.g., biomarker assessments) are important, they do not necessarily correlate with the perceived immune status (i.e., feelings of reduced resistance). Ultimately, only feelings of reduced resistance or feeling ill will elicit changes in health-related behavior, such as visiting a physician or taking other actions to improve one’s health (e.g., adopt a healthy lifestyle). Similarly, although sleep may be objectively disturbed, people are most likely to seek medical care when their subjectively experienced sleep complaints interfere with their daily activities. Therefore, it is essential to also acquire patient-related outcome measurements of perceived immune functioning and (subjective) sleep quality. Nevertheless, it is recommended that future prospective studies also include biomarker assessments (e.g., blood cytokine levels) to objectively assess the immune status as supportive evidence for the perceived immune functioning reported by the participants. A formal diagnosis of insomnia and impaired wound healing would further strengthen the design of future studies. It must be noted that, notwithstanding the subjective nature of the assessments in the current study, clear differences in sleep complaints and perceived immune fitness were found between the impaired wound healing groups and the control group. This observation gives confidence that our findings will be confirmed in more controlled studies in the future. Fourthly, some effect modifiers were not assessed in our study population. Examples of such variables are age, sex, smoking, or low physical activity. Studies described these factors as risk factors for the impaired healing of ulcers [65,66]. Sleep is also reported to be affected by nicotine, including difficulties initiating sleep, increased nonrestorative sleep, and difficulties waking up [67].

Finally, it remains to be determined to what extent other factors that were not assessed in the current study influence the observed relationship between insomnia complaints and poorer perceived immune fitness in individuals with impaired wound healing. An example of such a modifying factor may be stress experienced as part of student life [33]. Increases in the production of hormones such as corticosterone in response to stress [68,69] have been associated with delayed wound healing [70] and a reduced expression of proinflammatory cytokines (e.g., IL-1β) and growth factors [71]. Further research is needed to evaluate the impact of factors such as stress and their impact as a modulating factor in the association between sleep, immune fitness, and wound healing. Evaluations of the interventions to improve wound healing, for example, by improving sleep hygiene or nutritional or lifestyle interventions, are also warranted to improve the wellbeing and quality of life of patients suffering from chronic wounds.

## 5. Conclusions

Individuals with impaired wound healing reported poorer perceived immune functioning, increased insomnia complaints, daytime fatigue, and poorer sleep quality. These effects were statistically significant in the SHW and COMBI group. Since the assessments were based on self-reports and the sample comprised students, future studies should confirm these findings using objective assessments of sleep disorders (e.g., polysomnography), immune functioning (e.g., biomarkers) and formally diagnosed impaired wound healing, preferably in a larger sample also comprising non-students and older age groups.

## Figures and Tables

**Table 1:** Demographics.

Demographics	Control Group	WI Group	SHW Group	COMBI Group
*n*	1622	69	250	92
Sex (m/f)	270/1352	13/56	40/210	7/85
Age	21.3 (2.1)	21.3 (1.9)	21.2 (2.1)	21.0 (2.1)
Weight (kg)	67.0 (11.6)	68.3 (14.2)	67.0 (11.5)	65.2 (12.7)
Height (m)	1.73 (0.1)	1.72 (0.1)	1.72 (0.1)	1.72 (0.1)
BMI (kg/m^2^)	22.4 (3.2)	23.2 (3.5)	22.5 (3.3)	22.1 (3.3)
Perceived immune fitness	7.7 (1.3)	7.3 (1.4)	6.9 (1.5) *	6.9 (1.7) *

Significant differences from the control group (*p*-value adjusted for multiple pairwise comparisons, significant if *p* < 0.0083) are indicated by *. Abbreviations: *n* = number of subjects, m = male, f = female, BMI = body mass index, SHW = slow-healing wounds, WI = wound infection, and COMBI = combination of slow-healing wounds and wound infection.

**Table 2:** Sleep outcomes.

Sleep Outcomes	Control Group	WI Group	SHW Group	COMBI Group
Total sleep time	519.5 (64.9)	512.8 (63.2)	526.3 (70.6)	515.5 (61.3)
Sleep onset latency	21.4 (15.5)	20.4 (15.4)	23.9 (16.9)	23.5 (16.0)
Nightly awakenings	0.9 (1.0)	1.1 (1.3)	1.1 (1.1)	1.2 (1.4)
Sleep quality	7.0 (1.4)	6.9 (1.8)	6.5 (1.6) *‡	6.3 (1.8) *‡

Significant differences from the control group (*p*-value adjusted for multiple pairwise comparisons, significant if *p* < 0.0083) are indicated by *. Significant differences from the WI group are indicated by ‡. Abbreviations: SHW = slow-healing wounds, WI = wound infection, and COMBI = combination of slow-healing wounds and wound infection.

**Table 3:** SLEEP-50 insomnia scale item scores.

SLEEP-50 Insomnia Scale Items	Control Group	WI Group	SHW Group	COMBI Group
I have difficulty in falling asleep	1.9 (0.0)	1.9 (0.9)	2.1 (1.0) *	2.2 (1.0) *
Thoughts go through my head and keep me awake	2.0 (0.9)	2.0 (0.9)	2.3 (0.9) *	2.4 (1.0) *
I worry and find it hard to relax	1.9 (0.8)	2.1 (0.8) γ	2.2 (0.9) *	2.5 (0.9) *
I wake up during the night	1.8 (0.9)	2.0 (1.0)	2.1 (1.0) *	2.1 (1.0) *
After waking up in the night, I fall asleep slowly	1.5 (0.8)	1.6 (0.9)	1.7 (0.9) *	1.7 (1.0)
I wake up early and cannot get back to sleep	1.6 (0.9)	1.8 (1.0)	1.7 (0.9) *	1.9 (1.0) *
I sleep lightly	1.7 (0.9)	1.8 (1.0)	1.9 (1.0) *	2.0 (1.0) *
I sleep too little	2.0 (1.0)	2.1 (1.0)	2.2 (1.0)	2.3 (1.0)
Generally, I sleep badly	1.6 (0.8)	1.7 (1.0)	1.8 (1.0) *	1.9 (1.0) *
**Total insomnia score**	**16.0 (5.1)**	**16.9 (5.6)**	18.1 (5.9) *	19.1 (6.5) *
**Positive screen for insomnia (% ≥ 19)**	**25.8%**	45.2% *	31.9% *	44.6% *

Significant differences from the control group (*p*-value adjusted for multiple pairwise comparisons, significant if *p* < 0.0083) are indicated by *. Significant differences from the COMBI group are indicated by γ. Abbreviations: SHW = slow-healing wounds, WI = wound infection, and COMBI = combination of slow-healing wounds and wound infection.

**Table 4:** Daytime fatigue and vigor.

Daytime Fatigue and Vigor	Control Group	WI Group	SHW Group	COMBI Group
POMS—vigor, activity	9.4 (4.3)	8.7 (4.7)	8.0 (4.5) *	7.4 (4.1) *
POMS—fatigue	4.9 (4.7)	6.3 (5.1)	7.2 (6.0) *	7.7 (5.2) *

Significant comparisons with the control group (*p* < 0.0083) are indicated by *. No significant differences were found between the SHW and COMBI groups, between the WI and COMBI groups, or between the SHW and WI groups. Abbreviations: POMS = profiles of mood states, SHW = slow-healing wounds, WI = wound infection, and COMBI = combination of slow-healing wounds and wound infection.

**Table 5:** Correlations between perceived immune fitness and sleep outcomes.

Correlations with Perceived Immune Fitness	r	*p*-Value
SLEEP-50 insomnia score	−0.190	<0.001 *
Total sleep time	−0.058	0.011
Sleep onset latency	−0.122	<0.001 *
Nightly awakenings	−0.102	<0.001 *
Sleep quality	0.194	<0.001 *
POMS—vigor, activity	0.229	<0.001 *
POMS—fatigue	−0.260	<0.001 *

Spearman’s correlations and *p*-values are shown. Correlations are considered significant if *p* < 0.0071 (applying a Bonferroni’s correction for multiple correlations) and are indicated by *. Abbreviation: POMS = profiles of mood states.

## Data Availability

The data are available upon request from the corresponding author.
